# Usage of Mitogen-Activated Protein Kinase Small Molecule Inhibitors: More Than Just Inhibition!

**DOI:** 10.3389/fphar.2018.00098

**Published:** 2018-02-12

**Authors:** Steffen K. Meurer, Ralf Weiskirchen

**Affiliations:** Institute of Molecular Pathobiochemistry, Experimental Gene, and Clinical Chemistry, RWTH Aachen University, Aachen, Germany

**Keywords:** inhibitors, signal transduction, PDGF-BB, mitogen-activated protein kinases, SB203580, SP600125, PD98059, UO126

## Abstract

We have identified a phenomenon occurring in the usage of proposed “specific” Mitogen-activated protein kinase (MAPK) inhibitors. We found that especially inhibitors of p38 potentiate the activation of other MAPKs in various cell types. This finding will have tremendous impact on the interpretation of all former studies using MAPK inhibitors.

## Results

**Figure 1:**
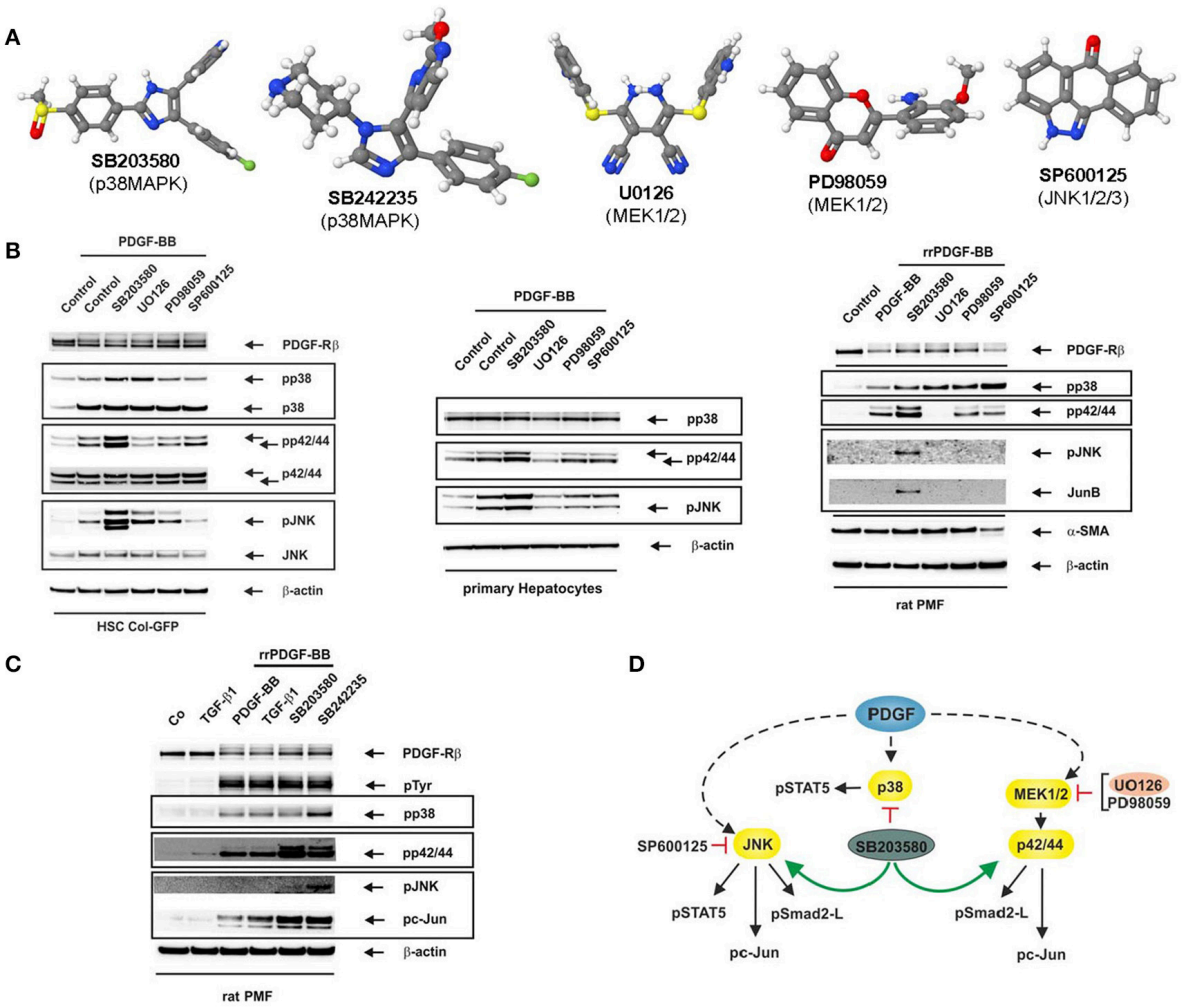
Reciprocal activation of MAPK signalling by MAPK inhibitors. **(A)** Images of inhibitors used in this study were generated with software Jmol (version 14.2.15). **(B)** The reporter cell line HSC Col-GFP (left), primary hepatocytes (middle) and (activated) PMF (right) were stimulated for 10 min with PDGF-BB (25 ng/ml) after pre-incubation of cells with the indicated inhibitors (each 10 μM) for 1 h. Thereafter, proteins were extracted and subjected to Western blot analysis with the depicted antibodies. The inhibition of MAP kinases impacts PDGF responses as PD98059 and UO126 reduce pp42/44 phosphorylation. In addition, SP600125 blunts c-Jun activation, while SB203580 and SB242235 reduce STAT5 phosphorylation (data not shown). **(C)** Rat PMF were stimulated for 10 min with TGF-β1 (1 ng/ml) or PDGF-BB (25 ng/ml) after pre-incubation of cells with the depicted p38 inhibitors (each 10 μM) for 1 h. **(D)** Deduced impact of inhibitors on MAP kinase activity in cultured HSC Col-GFP. Antibodies used are from Santa Cruz (PDGF-Rβ, sc-432), Cell Signaling (pp42/pp44, CS-9101; p42/p44, CS-4696; pSAPK/JNK, CS-9251; SAPK/JNK, CS-9252; pc-Jun, CS-9261; JunB, CS-3753), BD Biosciences (pp38, 612288; p38, 612168), Millipore (pTyr, 05-321), Cymbus Biotechnology (α-SMA, CBL 171), and Sigma (β-actin, A5441), respectively. In this scheme, PDGF stands for PDGF-BB.

Most of the Mitogen-activated protein kinase (MAPK) inhibitors have highly different structures (Figure 1A). The p38 MAP kinase inhibitors SB203580 and SB242235 (Lee et al., 1994; Ward et al., 2002) as well as SP600125 targeting JNK1, JNK2, and JNK3 (Bennett et al., 2001) are commonly used. In addition, the MEK inhibitors UO126 selective for MEK1 and MEK2 (Favata et al., 1998), and PD98059 primarily targeting MEK1 and MEK2 with a more than 10-fold lower affinity (Dudley et al., 1995) are established compounds which have been tested extensively (Davies et al., 2000; Bain et al., 2003). In hepatology, these inhibitors have significantly contributed to the knowledge in the field in which MAPKs contribute to inflammation, fibrogenesis, and hepatocellular carcinoma (Borkham-Kamphorst and Weiskirchen, 2016).

PDGF-BB is a potent mitogen for hepatic stellate cells (HSC) (Borkham-Kamphorst and Weiskirchen, 2016), and stimulation of HSC Col-GFP with PDGF-BB leads to activation of the three major MAP kinases (Figure 1B). As expected, the pre-treatment of cells with the MEK1/MEK2 inhibitors resulted in a direct reduction in ERK1/ERK2 MAPK phosphorylation, while SB203580 and SP600125 blunted MAPK activity as demonstrated by a reduction in substrate phosphorylation of STAT5 (p38, JNK) and c-Jun (JNK) (not shown).

Unexpectedly, blockade of p38 by SB203580 resulted in a significant increase in both ERK1/ERK2 and JNK phosphorylation. Likewise, the MEK1/2 inhibitors UO126 and PD98059 provoked increased phosphorylation of JNK and p38 (only UO126). Most sensitive to the application of small-molecule inhibitors was JNK that became activated by inhibitors targeting the p38 (SB203580) or ERK1/2 pathways. These results suggest that blocking of a MAP kinase by the corresponding inhibitor leads to a simultaneous activation of other MAPK-pathways driven by the same ligand. We found similar results in primary hepatocytes and primary (activated) portal myofibroblasts (PMF). In particular, these experiments revealed a strong stimulation of JNK and ERK phosphorylation in the presence of the p38 inhibitor SB203580. Moreover, the mutual “induction by inhibition” is also evident in PMF when the alternative p38 inhibitor SB242235 is used indicating that the finding is not an artefact of an individual inhibitor (Figure 1C). All experiments were highly reproducible (Supplementary Figure 1). In addition, we could show that not only MAPK phosphorylation itself but also substrate phosphorylation is increased which demonstrates a higher activity of non-targeted MAPKs (Supplementary Figure 2).

## Materials and methods

Isolation of primary cells (hepatocytes, PMF) and establishment of cell line HSC Col-GFP were done as described previously (Meurer et al., 2011, 2013; Borkham-Kamphorst et al., 2016). SDS-PAGE and Western blot analysis were done as reported (Borkham-Kamphorst et al., 2016).

## Discussion

The observation that a mutually “selective” MAPK-inhibitor becomes an activator of another MAPK-pathway physiologically stimulated by the same trigger has fundamental impact. Numerous reports have more or less uncritically applied MAPK inhibitors and concluded that a pathway targeted by a “specific” inhibitor is responsible for a biological effect. However, considering effects provoked by reciprocal activation loops challenge some of these studies. In our experimental setting, the influence of different small-molecule inhibitors resulted in dependencies depicted in Figure 1D.

It is obvious that the mutual “activation by inhibition” is not limited to straight forward MAPK-signaling network. Although we don't know if the phenomenon of cross-activation can be generalized when blocking one pathway, we think our observations must be critically kept in mind when interpreting experimental results mediated by a “specific” inhibitor.

Potential mechanisms of MAPK crosstalk and regulation by dual-specificity phosphatases under different conditions are discussed elsewhere (Birkenkamp et al., 2000; Shen et al., 2003; Junttila et al., 2008; Ríos et al., 2014).

## Bioethics

This study was carried out in accordance with the recommendation of the Landesamt für Umwelt und Naturschutz (LANUV, Recklinghausen, Germany). The protocols for isolation of primary cells were approved by the LANUV.

## Author contributions

RW and SM designed the study and drafted manuscript.

### Conflict of interest statement

The authors declare that the research was conducted in the absence of any commercial or financial relationships that could be construed as a potential conflict of interest.
