# Supplementary material for: Usage of Mitogen-Activated Protein Kinase Small Molecule Inhibitors: More Than Just Inhibition!
Source: Front Pharmacol. 2018 Feb 12;9:98. doi: 10.3389/fphar.2018.00098 (PMC5816342; doi:10.3389/fphar.2018.00098)
Supplement: Supplementary file 1 [file DataSheet1.DOC]

**Supplemental Figures**

**Suppl. Figure 1: Biological replicates of experiments depicted in Figure 1B and C.**

**Suppl. Figure 2: Testing substrate phosphorylation of non-targeted MAPKs.**
