# Supplementary figures and images for: Usage of Mitogen-Activated Protein Kinase Small Molecule Inhibitors: More Than Just Inhibition!
Source: Front Pharmacol. 2018 Feb 12;9:98. doi: 10.3389/fphar.2018.00098 (PMC5816342; doi:10.3389/fphar.2018.00098)

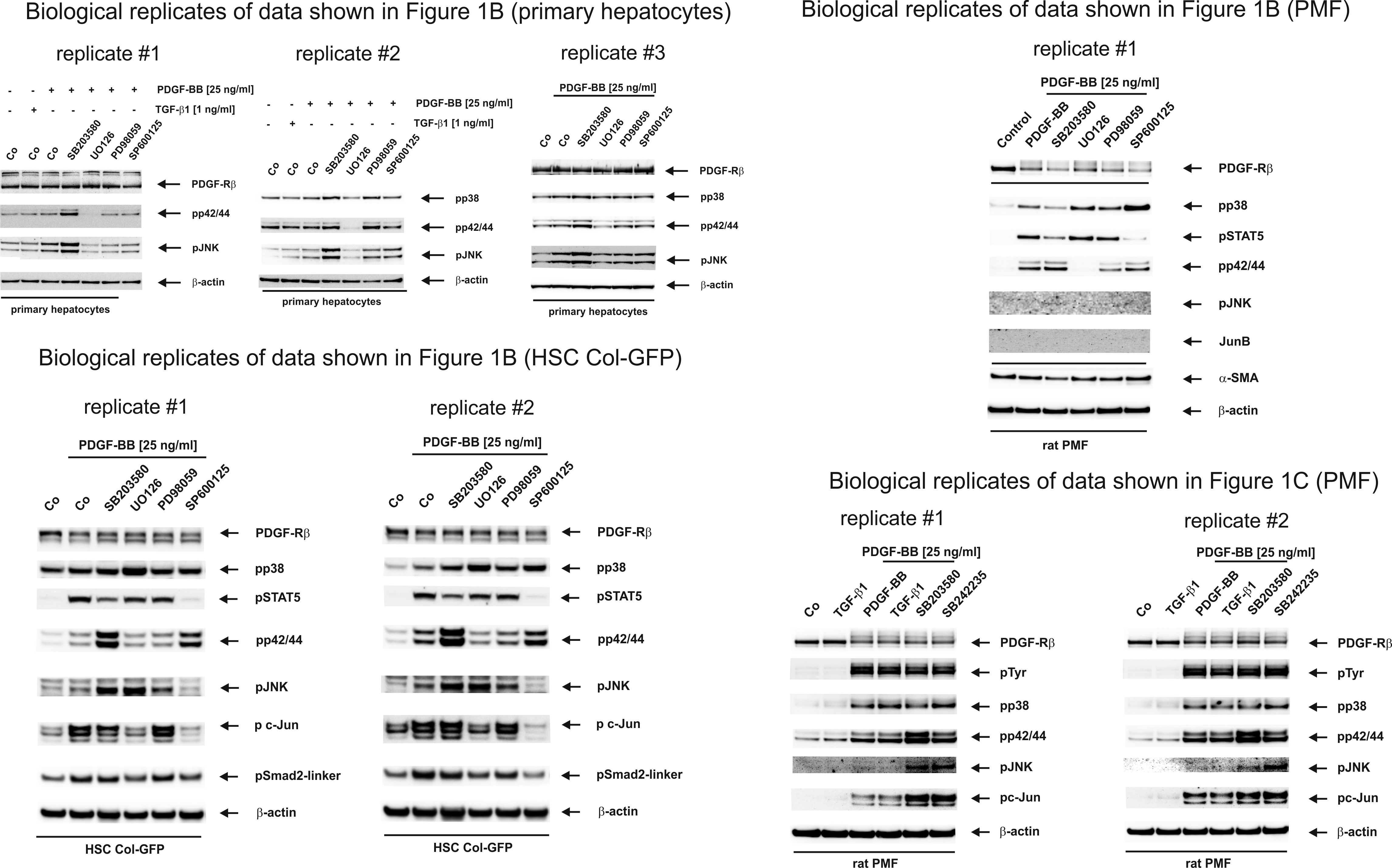

Supplement: Supplementary file 2 [file Image1.JPEG]

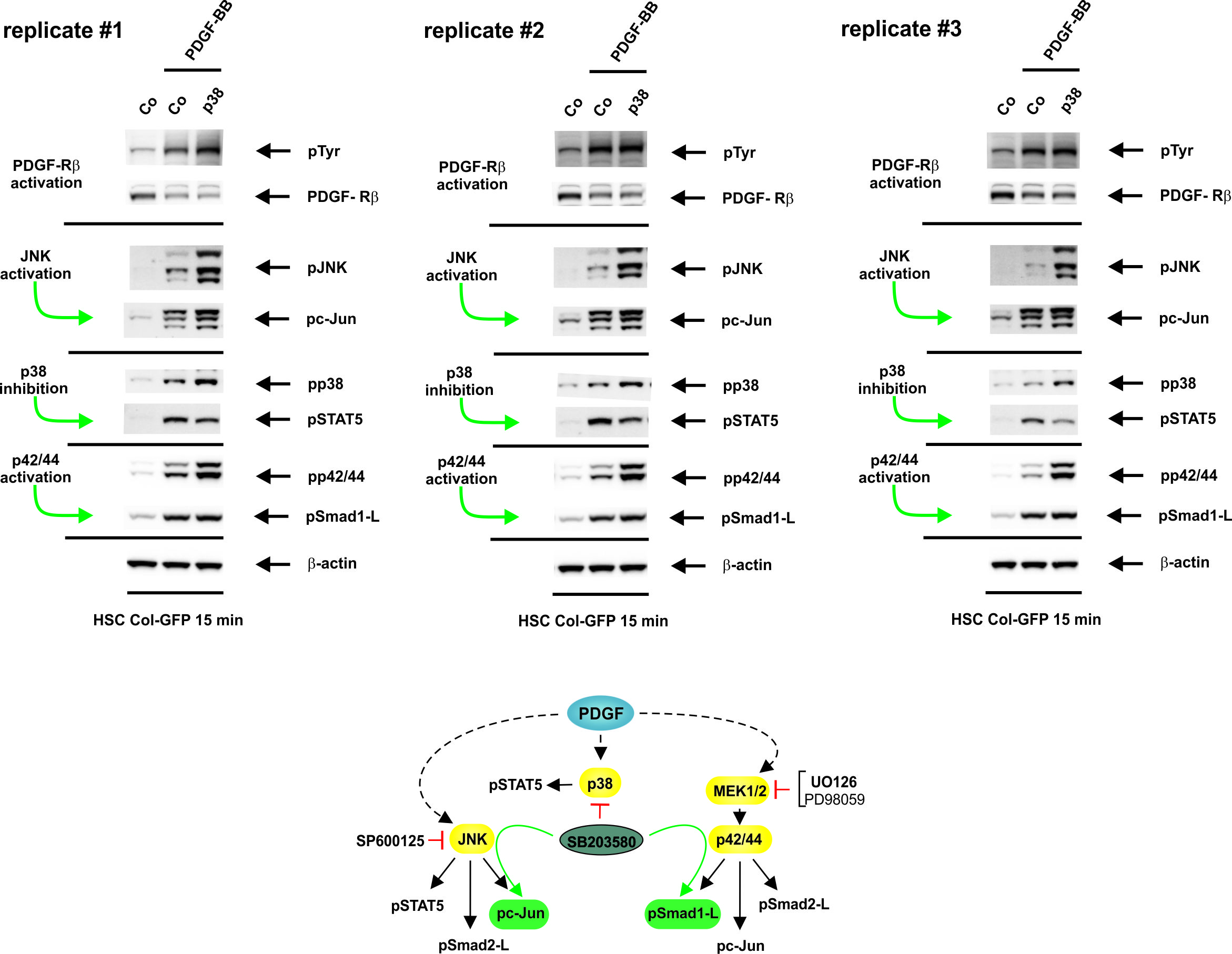

Supplement: Supplementary file 3 [file Image2.JPEG]
